# Social factors and suicidal ideation in adulthood

**DOI:** 10.1192/j.eurpsy.2021.995

**Published:** 2021-08-13

**Authors:** A. Rodríguez Lirón, S. Gayete, B. Olaya, J.L. Ayuso-Mateos, J. Haro

**Affiliations:** 1 Psychiatry, Parc Sanitari Sant Joan de Déu, Sant Boi de Llobregat, Spain; 2 Cibersam, Instituto de Salud Carlos III, Madrid, Spain; 3 Psychiatry, Universidad Autónoma de Madrid, Madrid, Spain; 4 Sant Boi De Llobregat, Parc Sanitari Sant Joan de Déu, Barcelona, Spain

**Keywords:** Suicidal ideation, age-related differences, loneliness, social support

## Abstract

**Introduction:**

In recent years, it has been possible to corroborate that people’s social environment is a key aspect in the study of suicide risk.

**Objectives:**

The aim of this study is to assess the relation between suicidal ideation and social factors (loneliness, social support, trust, participation and cohabiting) in a representative sample of the Spanish adult population, comparing the effect according to sex different age groups (18-49, 50-64, ≥65 years).

**Methods:**

Cross-sectional study of a representative sample of the Spanish population (n = 4,217) conducted between 2011 and 2012. Loneliness was assessed using the UCLA Loneliness Scale. Social support was assessed using the OSLO-3 Social Support Scale, and participation scale and trust. Data were analyzed using logistic regression models adjusting for sex, sociodemographic and health variables (lifestyles, depression, and multimorbidity).

**Results:**

Prevalence rates of suicidal ideation were higher in young and middle-aged adults. In the middle-aged groups, loneliness is significantly associated with suicidal ideation in both women and man. Among man, cohabiting and trust were identified as a protective factors of suicidal ideation. Among women, only social support was identified as a protective factor. In the older adult’s group, trust acted as a protective factor of suicide ideation among women. For man was the social support. Among younger adults, cohabiting was identified as a protective factor in man.

**Figure:**
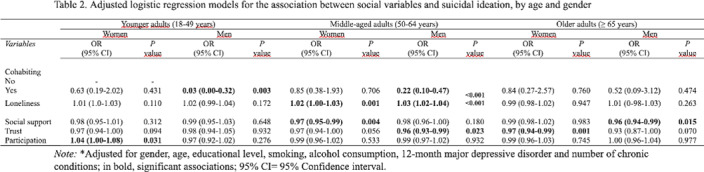

**Conclusions:**

Due to the different results involving social factors and suicidal ideation according to age and sex, we highlight the importance of studying social factors for the detection of specific needs among the Spanish adult population.

